# Maternal restrictive feeding and eating in the absence of hunger among toddlers: a cohort study

**DOI:** 10.1186/s12966-017-0630-8

**Published:** 2017-12-19

**Authors:** Katherine W. Bauer, Jess Haines, Alison L. Miller, Katherine Rosenblum, Danielle P. Appugliese, Julie C. Lumeng, Niko A. Kaciroti

**Affiliations:** 1 Department of Nutritional Sciences, University of Michigan School of Public Health, 1415 Washington Heights, Ann Arbor, MI 48109 USA; 2 Department of Family Relations and Applied Nutrition, Macdonald Stewart Hall, University of Guelph, Room, Guelph, ON 226 Canada; 3 Center for Human Growth and Development, University of Michigan, 300 North Ingalls Street, Ann Arbor, MI 48109 USA; 4 Department of Psychiatry, University of Michigan Medical School, Ann Arbor, MI 48109 USA; 5 North Easton, USA

**Keywords:** Feeding practices, Childhood obesity, Eating in the absence of hunger, Cohort study

## Abstract

**Background:**

Restrictive feeding by parents has been associated with greater eating in the absence of hunger (EAH) among children, a risk factor for obesity. However, few studies have examined the association between restrictive feeding and EAH longitudinally, raising questions regarding the direction of associations between restrictive feeding and child EAH. Our objective was to examine the bidirectional prospective associations between restrictive feeding and EAH among toddlers.

**Methods:**

Low-income mother-child dyads (*n* = 229) participated when children were 21, 27, and 33 months old. Restriction with regard to food amount and food quality were measured with the Infant Feeding Styles Questionnaire. EAH was measured as kilocalories of food children consumed after a satiating meal. A cross-lagged analysis adjusting for child sex and weight-for-length z-score was used to simultaneously test cross-sectional and bidirectional prospective associations between each type of restriction and children’s EAH.

**Results:**

At 21 months, mothers of children with greater EAH reported higher restriction with regard to food amount (*b* = 0.17, *p* < .05). Restriction with regard to food amount at age 21 months was inversely associated with EAH at 27 months (*b* = −0.20, p < .05). Restriction with regard to food amount at 27 months was not associated with EAH at 33 months and restriction with regard to food quality was not associated with EAH. EAH did not prospectively predict maternal restriction.

**Conclusions:**

Neither restriction with regard to food amount nor food quality increased risk for EAH among toddlers. Current US clinical practice recommendations for parents to avoid restrictive feeding, and the potential utility of restrictive feeding with regard to food amount in early toddlerhood, deserve further consideration.

**Electronic supplementary material:**

The online version of this article (10.1186/s12966-017-0630-8) contains supplementary material, which is available to authorized users.

## Background

Eating in the absence of hunger (EAH), or the extent to which a child continues to eat despite experiencing satiety [1], is an important behavioral phenotype indicating elevated risk of obesity. EAH, which is typically measured as the amount of highly-palatable snack foods that a child consumes after a satiating meal, has been associated with higher weight, weight gain, and obesity among children [2–7]. Although there is evidence that EAH is in part genetically determined [8], intervention studies suggest that EAH is modifiable [9, 10] and thus there is great interest in how sociocultural factors influence the development of EAH among children.

Much attention has been paid to how parenting practices may impact children’s obesogenic eating behavior, particularly EAH [11, 12]. Parents’ use of restrictive feeding practices has been hypothesized to promote EAH by undermining children’s ability to self-regulate their eating and increasing children’s desire for restricted foods [13, 14]. However, existing research regarding the association between restrictive feeding and EAH among children has produced mixed results. Early research among a primarily white, moderate socioeconomic status sample of girls found that between ages 5 and 7, the greatest increases in EAH were observed among overweight girls whose mothers reported higher levels of restrictive feeding [13]. Two additional cross-sectional studies among preschool-age children also found positive associations between maternal restriction and EAH among girls [1, 3]. Together, this body of work contributed to the current US practice guidelines for preventing and treating obesity among children, which encourage parents to avoid overly restricting children’s intake or restricting access to specific foods [15, 16]. Other studies of preschool-aged children however, have not observed any associations between restrictive feeding practices and EAH [17, 18]. Further, longitudinal data increasingly suggest that parents’ use of restrictive feeding practices is a response to, not a cause of, increasing child weight [19, 20]. In sum, the relations between restrictive feeding and child EAH are unclear.

Given the uncertainty regarding whether restrictive feeding contributes to EAH among children, the objective of the current study is to utilize longitudinal data from a contemporary cohort of toddlers to examine the bidirectional associations between maternal restriction and child EAH. All prior research on maternal restriction and child EAH has been conducted among children preschool age and older; no studies have been conducted among toddlers. However in the US, clinical recommendations for the prevention of childhood obesity advise parents of children 12 years and younger to avoid overly restrictive feeding [15], therefore it is essential to understand the potential impact of restrictive feeding among all ages of children in this range. Among the cohort of toddlers observed in the current study, EAH at 27 months predicted greater body mass index z-score at 33 months, demonstrating that EAH is a concerning behavior that emerges early in life and may establish risk of obesity throughout childhood [7]. These findings reinforce the urgency of identifying factors that contribute to EAH during toddlerhood.

## Methods

### Participants and recruitment

Participants were recruited between 2011 and 2014 via flyers posted in community agencies serving low-income families in Michigan, USA. The study was described to families as examining whether children with different levels of stress eat differently. Inclusion criteria were that the biological mother was the legal guardian, had an education level less than a 4-year college degree, and was at least 18 years old; the family was eligible for federally-funded nutrition, education, or health programs (e.g., Head Start, Women, Infants and Children (WIC) Program, or Medicaid), and was English-speaking; and the child was between 21 and 27 months old, was born at a gestational age ≥ 36 weeks, and had no food allergies or significant health problems, perinatal or neonatal complications, or developmental delays. Mothers provided written informed consent. The University of Michigan Institutional Review Board approved the study.

Mother-child dyads were invited to participate in data collection at ages 21, 27, and 33 months to capture early, middle, and late toddlerhood. At each age, the data collection procedures spanned across 5 days and included measures of eating behavior and biobehavioral self-regulation. A total of 244 dyads participated. Most (*n* = 186) dyads entered the study when the child was age 21 months, but 58 entered the study when the child was age 27 months to maximize recruitment. Measures obtained at study entry are henceforth referred to as “baseline” measures. The current study is limited to dyads who completed the feeding questionnaire at least once or participated at least once in the EAH protocol. A total of 229 of the 244 dyads met these requirements. The 229 dyads included in this analysis did not differ from the excluded dyads with regard to child sex, child age, child race/ethnicity, or maternal education. Including dyads who had completed the feeding questionnaire and/or the EAH protocol at least once in statistical analyses reduces the likelihood of selection bias as compared to only including participants with complete data at all time points [21]. A total of 81 children participated at only one age point, 86 participated at two age points, and 62 participated at three age points.

### Measures

Data collection was conducted in the dyads’ homes. Research assistants (*N* = 12 at 21 m, 10 at 27 m, and 8 at 33 m) were all bachelor’s degree-level study staff trained and certified in protocol administration. At all study time points, protocol scripts were provided and the research assistants were instructed to follow the scripts verbatim. To confirm that the research assistants were conducting the protocols with fidelity, the research assistants first observed the protocols being administered by a senior staff member and then administered the protocols themselves in the field under the guidance of a senior staff member. These assessment sessions were also videotaped and the research assistants were provided feedback on their protocol administration by one of the study’s principal investigators. Research assistants were allowed to administer the protocols without senior staff present after the project manager and principal investigators certified that they were able to conduct the protocols without needing any correction.

### Maternal restrictive feeding practices

We used two subscales from the Infant Feeding Styles Questionnaire (IFSQ) to measure mothers’ beliefs and behaviors regarding restriction with regard to (1) the amount of food children eat, and (2) the quality of food children eat. Items, presented in Additional file 1, are answered on a 5-point scale (1 to 5), with higher scores indicating more of the given behavior or belief, with reverse scoring applied as appropriate. Response options were “never”, “seldom”, “half of the time”, “most of the time”, and “always” for behaviors, and “disagree”, “slightly disagree”, “neutral”, “slightly agree”, and “agree” for beliefs. In original validation testing, the subscales had moderate to high internal consistency (H coefficient = 0.75 for restriction with regard to amount and 0.85 for restriction with regard to food quality) [22]. Among the current study sample, subscales also had moderate to high internal consistency: restriction with regard to food amount (4 items; α = 0.64–0.70 across ages) and restriction with regard to food quality (7 items; α = 0.73–0.77 across ages).

### Eating in the absence of hunger protocol

Mother-child dyads participated in a standardized protocol [1] to assess the child’s EAH at each age. Mothers were asked to have their children fast for 1 h and then serve a typical lunch that included at least two different foods and one drink. Mothers were instructed to make enough food for the child’s lunch such that the child would likely leave food on their plate. When the child finished all the food offered or did not eat more when their mother provided more food, the research assistant confirmed the child’s fullness by asking the mother, “Would you say your child is done eating?” If the child did not finish the initial serving of food and/or seemed uninterested in the food, the research assistants asked the mother, “Would you say your child is still hungry or would you say that your child is done eating?” If a mother’s response to either question suggested her child was still hungry, the child was given more time to eat and/or provided additional food.

After the lunch ended, the research assistant presented a standardized plate of highly-palatable sweet and salty snack foods (Table 1) and told the child, “Here are some special treats you can eat.” Foods selected for the standardized plate are commonly eaten by children in the United States, are highly palatable, and are not choking hazards for children in this age range. Mothers also reported how often the child had eaten the food in the past 4 weeks. To signal to the toddler that they could eat the foods offered [23–26], the experimenter ate one Oreo cookie off the plate and said, “I’m going to have one, too. Mmm this is really good. You can eat as much as you want.” The child was then given free access to the food. The mother was asked not to interact with the child during the protocol to minimize external prompts to eat. After 10 min, the plate of food was removed. Remaining food was weighed and kilocalories (kcal) of each food consumed were calculated.

**Table 1:** Foods presented in Eating in the Absence of Hunger Protocol

Food	Serving	Weight (grams) per serving, mean (SD)	Kilocalories per serving, mean (SD)	Frequency of eating in last 4 weeks^a^, mean (SD)		
				21 months	27 months	33 months
Sweet Foods						
Nabisco Original Chips Ahoy chocolate chip cookies	2 cookies	22.0 (0.7)	106.4 (3.5)	1.0 (1.1)	1.0 (1.1)	0.7 (0.7)
Nabisco Original Oreo cookies	2 cookies	23.2 (0.8)	109.3 (3.9)	1.0 (1.0)	0.9 (0.9)	0.8 (0.7)
Keebler Animal Cookies, Frosted	5 cookies	19.0 (1.5)	97.9 (7.8)	0.2 (0.7)	0.2 (0.5)	0.2 (0.5)
Nabisco Rainbow Candy Blast Chips Ahoy cookies	2 cookies	33.4 (1.2)	176.8 (6.4)	0.1 (0.4)	0.2 (0.5)	0.2 (0.4)
Kellogg’s Keebler Fudge Stripe chocolate-coated cookies	2 cookies	23.6 (3.4)	122.1 (17.7)	0.2 (0.5)	0.2 (0.4)	0.2 (0.5)
Salty Foods						
Pringles potato chips	10 chips	18.2 (0.7))	97.6 (3.6)	1.6 (1.2)	1.7 (1.2)	1.6 (1.1)
Frito-Lay Cheetos cheese puffs	10 puffs	20.3 (3.3)	108.8 (17.5)	1.0 (1.3)	1.1 (1.1)	0.8 (1.0)

^a^Response options: 0 = Never; 1 = 1–3 times in the past 4 weeks; 2 = 1×/wk.; 3 = 2-4×/wk.; 4 = 5-6×/wk.; 5 = 1×/day; 6 = 2-3×/day; 7 = 4-5×/day; 8 = ≥6×/day

### Anthropometry

Children’s weight and length were measured by trained research staff. Weight-for-length z-score (WLZ) was calculated based on the US Centers for Disease Control Growth Charts [27]. For descriptive purposes, we categorized children as obese (WLZ ≥ 95th percentile for age and sex), overweight (WLZ ≥ 85th percentile and <95th percentile), normal weight (WLZ <85th percentile and >5th percentile), and underweight (WLZ ≤ 5th percentile). Mothers’ weight and height were measured and BMI calculated. Mothers were categorized as obese (BMI ≥ 30), overweight (BMI ≥ 25 and <30), normal weight (BMI < 25 and >18.5), and underweight (BMI ≤ 18.5).

### Sociodemographic characteristics

Mothers reported sociodemographic characteristics for herself and her child including the child’s birthdate, sex, and race and ethnicity, and her own educational attainment.

### Statistical analysis

**Fig. 1:**
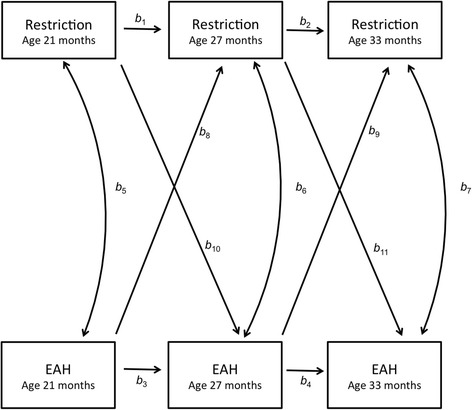
Path model of concurrent and cross-lagged associations between maternal restriction and child eating in the absence of hunger (EAH) between 21, 27, and 33 months

Univariate statistics were used to describe the sample. Mixed effects models were used to examine differences in mean EAH kcal and mean maternal restriction across the three age points. Cross-lagged path models were fitted using MPLUS version 4.1 (Muthen & Muthen, Los Angeles, CA) to test the association between maternal restriction and EAH at ages 21, 27, and 33 months (Fig. 1), adjusting for child sex and weight-for-length z-score. Cross-lagged path models allow for simultaneously estimating three types of associations: longitudinal associations between the same measures over time, for example, maternal restriction at 21 months and maternal restriction at 27 months; concurrent correlations between maternal restriction and child EAH at each age point; and cross-lagged associations simultaneously estimating the effect of maternal restriction at an earlier age with child EAH at a later age, and vice versa, elucidating the direction of association between these two constructs. Bayesian estimation technique in MPLUS was used to fit these models. Missing data were handled using a Bayesian approach using appropriate posterior distribution. The posterior distribution was constructed based on a full information likelihood with non-informative prior distribution for parameters of interest and was used to derive inferences in path analysis, where each path was estimated based on all available data for that path. If a dyad only participated at one time point, their data only contributed to the estimations of the mean and variance at that time point. If a dyad had longitudinal data available, their data contributed to the estimation of a longitudinal path. The Bayesian approach using the full information likelihood provides valid estimates under a missing at random missing-data mechanism, which is less restrictive than the data missing completely at random [28]. Bayesian posterior predictive checks (ppc) using Chi-square statistics and the corresponding posterior predictive *p*-values (ppp) were used to assess the goodness of fit in each model. A ppp value within 0.05 to 0.95 range indicates acceptable fit for the model [29].

## Results

The child sample was 52.4% male and 45.0% were non-Hispanic white (Table 2). Among children, 16.5% were overweight and 14.6% had obesity. Among mothers, 37.6% reported that some high school or graduating high school/receiving a GED was their highest level of educational attainment. Over half of mothers (53.6%) had obesity and 18.7% were overweight. Mean EAH increased as children aged, from 89.7 kcal at 21 months, to 105.2 kcal at 27 months, to 123.2 kcal at 33 months (*p* < .001) (Table 3). Mothers’ restriction with regard to food amount and with regard to food quality were not significantly different between the different age points (*p* = .54 and *p* = .15, respectively). The two restriction subscales were not correlated with each other at any of the study time points (Additional file 2).

**Table 2:** Characteristics of the children and mothers at baseline (*N* = 229)

	% (n) or Mean (Standard Deviation)
Child Sex	
Female	47.6 (109)
Male	52.4 (120)
Child Race/Ethnicity	
Non-Hispanic white	45.0 (103)
Hispanic or non-white	55.0 (126)
Child weight status	
Underweight	3.0 (5)
Normal weight	65.9 (108)
Overweight	16.5 (27)
Obese	14.6 (24)
Maternal weight status	
Underweight	1.3 (2)
Normal weight	26.5 (41)
Overweight	18.7 (29)
Obese	53.6 (83)
Maternal Education	
High school graduate/GED or less	37.6 (86)
Some college or completed 2-year degree	62.5 (143)

**Table 3:** Child eating in the absence of hunger and maternal restriction by time period

	21 months	27 months	33 months	*p*-value
Kilocalories consumed by child during eating in the absence of hunger protocol				
Mean (SD)	89.7 (53.1)	105.2 (65.6)	123.2 (71.3)	<.001
Interquartile Range	56.9–113.5	60.6–147.7	65.3–167.9	
Maternal restriction with regard to food amount				
Mean (SD)	3.3 (1.1)	3.2 (1.1)	3.1(1.0)	.54
Interquartile Range	2.5–4.0	2.5–4.0	2.5–3.8	
Maternal restriction with regard to diet quality				
Mean (SD)	3.1 (0.8)	3.1 (0.8)	3.1 (0.8)	.15
Interquartile Range	2.7–3.6	2.6–3.7	2.6–3.6	

As depicted in Fig. 1, the results of the cross-lagged analysis demonstrate the simultaneously-adjusted longitudinal associations between the same measures over time, concurrent correlations between maternal restriction and child EAH at each age point, and cross-lagged associations estimating the effect of maternal restriction at an earlier age with child EAH at a later age, and vice versa. The fit of both cross-lag models (Model 1: Restriction with regard to food amount and Model 2: Restriction with regard to food quality) was good with the ppp value for each model well within the recommended 0.05 to 0.95 range.

In Model 1, mothers’ restriction with regard to food amount was strongly correlated between 21 and 27, and 27 and 33 months (*b*_*1*_ *=* 0.62 and *b*_*2*_ *=* 0.60, respectively, *p* < .05; Table 4). Children’s measures of EAH were not significantly correlated between 21 and 27 months (*b*_*3*_ = 0.19, *p* > .05) but were significantly correlated between 27 and 33 months (*b*_*4*_ *=* 0.46, *p* < .05), after adjustment for concurrent and longitudinal associations with maternal restriction with regards to food amount. Maternal restriction with regard to food amount at 21 months was positively associated with child EAH at 21 months (*b*_*5*_ = 0.17, *p* < .05) and was inversely associated with EAH at 27 months (*b*_*10*_ = −0.20, *p* < .05). However, maternal restriction with regard to food amount at 27 months was not associated with child EAH at 33 months (*b*_*11*_ = 0.03, *p* > .05).

**Table 4:** Path coefficients for model shown in Fig. 1 in total sample (*n* = 229)

		Path										
		Longitudinal associations of restriction		Longitudinal associations of EAH		Concurrent associations of Restriction and EAH			EAH predicting future Restriction		Restriction predicting future EAH	
		Restriction 21 m → Restriction 27 m	Restriction 27 m → Restriction 33 m	EAH 21 m → EAH 27 m	EAH 27 m → EAH 33 m	Restriction 21 m → EAH 21 m	Restriction 27 m → EAH 27 m	Restriction 33 m → EAH 33 m	EAH 21 m → Restriction 27 m	EAH 27 m → Restriction 33 m	Restriction 21 m → EAH 27 m	Restriction 27 m → EAH 33 m
		*b*_1_	*b*_2_	*b*_3_	*b*_4_	*b*_5_	*b*_6_	*b*_7_	*b*_8_	*b*_9_	*b*_10_	*b*_11_
Model 1	Restriction with regard to food amount ppp = 0.14	0.62^*^	0.60^*^	0.19	0.46^*^	0.17^*^	−0.02	0.01	−0.07	0.01	−0.20^*^	0.03
Model 2	Restriction with regard to food quality ppp = 0.23	0.68^*^	0.59^*^	0.14	0.47^*^	−0.03	−0.09	−0.08	0.01	0.13	0.05	0.08

Note: EAH = eating in the absence of hunger; ppp = posterior predictive *p*-values; ^*^ *p* < .05

In Model 2, mothers’ restriction with regard to food quality was strongly correlated between 21 and 27, and 27 and 33 months (*b*_*1*_ *=* 0.68 and *b*_*2*_ *=* 0.59, respectively, p < .05). Similar to Model 1, in Model 2 adjusting for concurrent and longitudinal associations with maternal restriction with regard to food quality, a non-significant correlation between measures of child EAH was observed between 21 and 27 months (*b*_*3*_ *=* 0.14, *p* > .05) while a significant correlation was observed between 27 and 33 months (*b*_*4*_ *=* 0.47, *p* < .05).

Maternal restriction with regard to food quality was not concurrently associated with child EAH at any time point (*b*_*5*_ *=* −0.03 at 21 months, b_6_ = −0.09 at 27 months, and b_7_ = −0.08 at 33 months, all *p* > .05) and did not prospectively predict child EAH at either of the future age points (b_10_ = 0.05 and b_11_ = 0.08, *p* > .05).

Child EAH did not prospectively predict either type of maternal restriction at the future age points (Model 1: b_8_ = −0.07 and b_9_ = 0.01, *p* > .05; Model 2: b_8_ = 0.01 and b_9_ = 0.13, *p* > .05).

## Discussion

The objective of this study was to examine the bidirectional associations between maternal restrictive feeding and EAH among toddlers between ages 21 and 33 months. Cross-lagged modeling of longitudinal data, as conducted in this and other recent studies [20, 30], can provide important clarity to the bidirectional relations inherent in child feeding. Contrary to research conducted primarily among preschool and school age children that suggests that restrictive feeding increases EAH among children [1, 3, 13], we observed an inverse association between restriction with regard to food amount at 21 months and EAH at 27 months. Within this cohort, EAH at 27 months was predictive of higher BMI z-score at 33 months [7], suggesting that lower restriction with regard to food amount in early toddlerhood may contribute to obesogenic eating during a critical developmental period. Restriction with regard to food amount at 27 months was unrelated to EAH at 33 months and restriction with regard to food quality did not prospectively predict EAH. These findings extend previous cross-sectional research that observed no associations between maternal restriction and EAH during the preschool period [17, 18]. While at 21 months, mothers of children with high EAH were more likely to report restriction with regard to food amount, prospectively, child EAH did not predict changes in mothers’ restrictive practices. This suggests that mothers of young children may not consistently recognize eating in the absence of hunger or be concerned enough about their children’s over eating at this young age to modify their feeding practices in response [31]. Mothers’ beliefs about the amount of food toddlers should eat or the types of food toddlers should eat may also be more strongly influenced by external sources, such as the media or clinicians, as opposed to characteristics of their child. Together, these and other findings [19, 20] suggest that current practice guidelines that pediatric providers should counsel parents to avoid overly restrictive feeding practices [15, 16] may be unfounded. While as a whole, maternal restriction appears to have little impact on children’s EAH, mothers’ efforts to limit the quantity of food that young toddlers eat may protect against obesogenic eating.

Differences between the current study and seminal studies suggesting that restrictive feeding increases EAH [1, 3, 13] may serve to explain our contrary findings. First, all previous research measured restrictive feeding practices used either the Child Feeding Questionnaire (CFQ) [32] or less commonly, the Comprehensive Feeding Practices Questionnaire (CFPQ) [33], while the IFSQ was used in the current study. The IFSQ may capture sufficiently different approaches to or perspectives on restriction as compared to other measures. Further, the IFSQ distinguishes between restriction with regard to food quality and restriction with regard to food amount. Most items in the CFQ, the most commonly-used measure of maternal restriction, ask about restriction of children’s “favorite foods”, junk foods, and sweets. Therefore, the CFQ restriction scale is more similar to the IFSQ’s restriction with regard to food quality subscale than the restriction with regard to food amount subscale. The lack of items specifically tapping into restriction with regard to food amount in the CFQ may serve to explain why previous studies using the CFQ have not observed that restriction may be protective against EAH.

Children in our sample were also younger than those in early studies of restriction [1, 3, 13]. While maternal restriction among older children may be detrimental, restrictive feeding does not appear to be detrimental to young children’s intake. This discrepancy by age of study population may be due to differences in the cognitive development of toddlers versus older children, or differences in the ways mothers restrict children of different ages. For example, mothers of older children may use more overt approaches to restriction [34] and/or older children may be more aware than younger children that their intake is being restricted.

Finally, sociodemographic and secular differences in study populations may explain differences between the current study’s findings and those of early studies. Original research on restrictive feeding was conducted over 15 years ago among primarily normal-weight samples, often exclusively girls from moderate-income families [1, 3, 13]. Our contemporary sample consisted exclusively of low-income children, many of whom were already overweight or obese by age 2. In the US, 40% of children live in low-income households [35]. These children are at substantially higher risk for overweight and obesity in early childhood [36], therefore identifying factors that contribute to the early emergence of obesogenic eating among this population is a priority. However, low-income children may be differently influenced by maternal restriction than higher income children. For example, low-income children are more likely than higher income children to live in environments where large portions of highly-palatable food are frequently available [37, 38]. In such environments, maternal restriction of food amount may be helpful to set children’s expectations of appropriate intake. In comparison, for children from higher income families where unhealthy food is less commonly available in their environment, maternal restriction may be experienced as more severe or limiting, resulting in increased desire for those foods. Given this potential for a differential impact of restrictive feeding on child eating by socioeconomic status, further research is needed with diverse study samples to inform clinical guidelines.

The lack of increase in EAH among children exposed to higher maternal restriction with regard to food amount has important implications for the development of obesity prevention interventions and clinical guidance targeting families. Serving portion sizes that lead to appropriate growth patterns may help to entrain children’s recognition of and response to satiety cues linked with these portion sizes. This perspective supports current obesity prevention practice guidelines that encourage parents to limit children’s portion sizes [15]. Previous research has also demonstrated that serving age-appropriate portion sizes limits the energy consumed by children during eating occasions when parents are present [39]. Our findings extend this research by suggesting that these portion size limits may also positively impact consumption during future eating occasions when children’s portion sizes are not under the control of parents. The current study additionally suggests that restricting children’s consumption of specific types of food, in particular foods of low nutritional quality that are high in added sugar and/or solid fats (e.g., candy, cookies, potato chips), does not increase children’s consumption of these types of foods when children are provided unrestricted access. This finding further supports current obesity prevention guidelines that recommend limiting children’s intake of energy dense, low nutritional quality foods [15], and provides evidence that restriction may not increase young children’s future consumption of these highly-palatable foods.

There are several limitations to this study to consider. The longitudinal design is a strength, but due to the high-risk nature of the study cohort, attrition was high and there were missing data, which affected study power. Missing data were handled using the recommended method—a Bayesian approach using appropriate posterior distribution to account for the missing data. This approach, versus listwise deletion of participants who were missing data at any time point, protects against inducing selection bias [40]. All the data available at each time period were used in the path analysis. That is, if a dyad only participated at one time point, their data only contributed to the estimations of the mean and variance at that time point. If a dyad had longitudinal data available, their data contributed to the estimation of a longitudinal path. Specifically, 167 dyads contributed information at 21 months, 186 dyads at 27 months, and 161 dyads at 33 months. With regard to the longitudinal paths, 125 dyads contributed information to the paths between 21 and 27 months and 149 dyads contributed to the paths between 27 and 33 months. Our examination of attrition supports the use of this strategy as there were few differences in families who participated in a single versus multiple data collections. Another study limitation is that the EAH protocol used differed slightly from the more commonly-used EAH protocols published in the literature. For example, in this study, data collection was conducted in dyads’ homes and children were not provided a standardized meal before having free access to the snack foods. However, there is similar variation across other studies of EAH in factors such as location of protocol administration and types of foods served as the preload meal [41]. Standardizing the EAH protocol for use across varying study populations may increase consistency and reproducibility within the scientific literature. Finally, similar to other measures of restrictive feeding, the IFSQ captures both mothers’ restrictive practices and mothers’ beliefs with regard to restriction. Future longitudinal research is needed to identify whether specific restrictive practices increase children’s risk for, or protect against, EAH.

## Conclusion

The current study identified that among low-income toddlers, restrictive feeding was not a risk factor for EAH. This suggests that interventions to improve children’s diet quality through limiting non-nutritive, energy-dense foods, as is recommended by current pediatric obesity prevention guidelines, are not detrimental to future eating behavior. Restriction with regard to food amount in particular may be protective against increasing EAH during early toddlerhood. Limiting toddlers’ intake to age-appropriate portions may help children appropriately recognize hunger and satiety signals as they age, and promote self-regulation of eating in situations where parents are not in control of children’s intake. Our results call into question the appropriateness of discouraging restrictive feeding, particularly among young children at high risk for obesity. Findings suggest that there may be appropriate approaches to restrictive feeding at specific points in children’s development. Future work is needed to identify the most effective parental feeding approaches that promote healthy weight throughout children’s development.

## Additional files

Additional file 1: Infant Feeding Styles Questionnaire sub-scale items. (DOCX 11 kb)

Additional file 2: Unadjusted Pearson correlation coefficients for maternal restrictive feeding and children’s eating in the absence of hunger at 21, 27, and 33 months. (DOCX 55 kb)

## Abbreviations

BMI: Body mass index
EAH: eating in the absence of hunger
GED: General Educational Development
IFSQ: Infant Feeding Styles Questionnaire
kcal: kilocalories
SE: standard error
WIC: Women Infants and Children
WLZ: Weight-for-length z-score

## References

[1] Fisher JO, Birch LL (1999). Restricting access to foods and children's eating. Appetite.

[2] Butte NF, Cai G, Cole SA, Wilson TA, Fisher JO, Zakeri IF, Ellis KJ, Comuzzie AG (2007). Metabolic and behavioral predictors of weight gain in Hispanic children: the viva la Familia study. Am J Clin Nutr.

[3] Faith MS, Berkowitz RI, Stallings VA, Kerns J, Storey M, Stunkard AJ (2006). Eating in the absence of hunger: a genetic marker for childhood obesity in prepubertal boys?. Obesity.

[4] Fisher JO, Birch LL (2002). Eating in the absence of hunger and overweight in girls from 5 to 7 y of age. Am J Clin Nutr.

[5] Hill C, Llewellyn CH, Saxton J, Webber L, Semmler C, Carnell S, van Jaarsveld CH, Boniface D, Wardle J (2008). Adiposity and ‘eating in the absence of hunger’ in children. Int J Obes.

[6] Kral TV, Allison DB, Birch LL, Stallings VA, Moore RH, Faith MS (2012). Caloric compensation and eating in the absence of hunger in 5- to 12-y-old weight-discordant siblings. Am J Clin Nutr.

[7] Asta K, Miller AL, Retzloff L, Rosenblum K, Kaciroti NA, Lumeng JC (2016). Eating in the absence of hunger and weight gain in low-income toddlers. Pediatrics.

[8] Fisher JO, Cai G, Jaramillo SJ, Cole SA, Comuzzie AG, Butte NF (2007). Heritability of hyperphagic eating behavior and appetite-related hormones among Hispanic children. Obesity.

[9] Boutelle KN, Zucker N, Peterson CB, Rydell S, Carlson J, Harnack LJ (2014). An intervention based on Schachter's externality theory for overweight children: the regulation of cues pilot. J Pediatr Psychol.

[10] Boutelle KN, Zucker NL, Peterson CB, Rydell SA, Cafri G, Harnack L (2011). Two novel treatments to reduce overeating in overweight children: a randomized controlled trial. J Consult Clin Psychol.

[11] Vaughn AE, Ward DS, Fisher JO, Faith MS, Hughes SO, Kremers SP, Musher-Eizenman DR, O'Connor TM, Patrick H, Power TG (2016). Fundamental constructs in food parenting practices: a content map to guide future research. Nutr Rev.

[12] Savage JS, Fisher JO, Birch LL (2007). Parental influence on eating behavior: conception to adolescence. J Law Med Ethics.

[13] Birch LL, Fisher JO, Davison KK (2003). Learning to overeat: maternal use of restrictive feeding practices promotes girls’ eating in the absence of hunger. Am J Clin Nutr.

[14] Jansen E, Mulkens S, Jansen A (2007). Do not eat the red food!: prohibition of snacks leads to their relatively higher consumption in children. Appetite.

[15] Barlow SE (2007). Expert committee recommendations regarding the prevention, assessment, and treatment of child and adolescent overweight and obesity: summary report. Pediatrics.

[16] Gidding SS, Dennison BA, Birch LL, Daniels SR, Gillman MW, Lichtenstein AH, Rattay KT, Steinberger J, Stettler N, Van Horn L (2006). Dietary recommendations for children and adolescents: a guide for practitioners. Pediatrics.

[17] Blissett J, Haycraft E, Farrow C (2010). Inducing preschool children's emotional eating: relations with parental feeding practices. Am J Clin Nutr.

[18] Harris H, Mallan KM, Nambiar S, Daniels LA (2014). The relationship between controlling feeding practices and boys’ and girls’ eating in the absence of hunger. Eat Behav.

[19] Rhee KE, Coleman SM, Appugliese DP, Kaciroti NA, Corwyn RF, Davidson NS, Bradley RH, Lumeng JC (2009). Maternal feeding practices become more controlling after and not before excessive rates of weight gain. Obesity.

[20] Derks IP, Tiemeier H, Sijbrands EJ, Nicholson JM, Voortman T, Verhulst FC, Jaddoe VW, Jansen PW (2017). Testing the direction of effects between child body composition and restrictive feeding practices: results from a population-based cohort. Am J Clin Nutr.

[21] Little RJ, Rubin DB. Statistical analysis with missing data: Wiley; 2014.

[22] Thompson AL, Mendez MA, Borja JB, Adair LS, Zimmer CR, Bentley ME (2009). Development and validation of the infant feeding style questionnaire. Appetite.

[23] Cashdan E (1994). A sensitive period for learning about food. Hum Nat.

[24] Harper LV, Sanders KM (1975). The effect of adults’ eating on young children’s acceptance of unfamiliar foods. J Exp Child Psychol.

[25] Hendy H, Raudenbush B (2000). Effectiveness of teacher modeling to encourage food acceptance in preschool children. Appetite.

[26] Highberger R, Carothers L (1977). Modification of eating behavior of toddlers in a day care setting. Fam Consum Sci Res J.

[27] Kuczmarski RJOC, Guo SS (2002). 2000 CDC growth charts for the United States: methods and development. National Center for Health Statistics. Vital Health Stat.

[28] Enders CK (2001). The impact of nonnormality on full information maximum-likelihood estimation for structural equation models with missing data. Psychol Methods.

[29] Gelman A (2004). Bayesian data analysis.

[30] Steinsbekk S, Barker ED, Llewellyn C, Fildes A, Wichstrom L. Emotional feeding and emotional eating: reciprocal processes and the influence of negative affectivity. Child Dev. 2017; 10.1111/cdev.12756 28439888

[31] Pesch MH, Rizk M, Appugliese DP, Rosenblum KL, Miller A, Lumeng JC (2016). Maternal concerns about children overeating among low-income children. Eat Behav.

[32] Birch LL, Fisher JO, Grimm-Thomas K, Markey CN, Sawyer R, Johnson SL (2001). Confirmatory factor analysis of the child feeding questionnaire: a measure of parental attitudes, beliefs and practices about child feeding and obesity proneness. Appetite.

[33] Musher-Eizenman D, Holub S (2007). Comprehensive feeding practices questionnaire: validation of a new measure of parental feeding practices. J Pediatr Psychol.

[34] Ogden J, Reynolds R, Smith A (2006). Expanding the concept of parental control: a role for overt and covert control in children's snacking behaviour?. Appetite.

[35] Jiang Y, Ekono M, Skinner C (2016). Basic facts about low-income children: children under 18 years, 2014. In*.* Edited by poverty NCfCi.

[36] Datar A, Chung PJ (2015). Changes in socioeconomic, racial/ethnic, and sex disparities in childhood obesity at school entry in the United States. JAMA Pediatr.

[37] Ding D, Sallis JF, Norman GJ, Saelens BE, Harris SK, Kerr J, Rosenberg D, Durant N, Glanz K. Community food environment, home food environment, and fruit and vegetable intake of children and adolescents. J Nutr Educ Behav. 2011; 10.1016/j.jneb.2010.07.003 21531177

[38] Larson NI, Story MT, Nelson MC (2009). Neighborhood environments: disparities in access to healthy foods in the U.S. Am J Prev Med.

[39] Johnson SL, Hughes SO, Cui X, Li X, Allison DB, Liu Y, Goodell LS, Nicklas T, Power TG, Vollrath K (2014). Portion sizes for children are predicted by parental characteristics and the amounts parents serve themselves. Am J Clin Nutr.

[40] Rubin DB. Inference and missing data. Biometrika. 1976:581–92.

[41] Lansigan RK, Emond JA, Gilbert-Diamond D (2015). Understanding eating in the absence of hunger among young children: a systematic review of existing studies. Appetite.

