# Supplementary material for: Maternal restrictive feeding and eating in the absence of hunger among toddlers: a cohort study
Source: Int J Behav Nutr Phys Act. 2017 Dec 19;14:172. doi: 10.1186/s12966-017-0630-8 (PMC5735902; doi:10.1186/s12966-017-0630-8)
Supplement: Supplementary file 1 — Infant Feeding Styles Questionnaire sub-scale items. (DOCX 11 kb) [file 12966_2017_630_MOESM1_ESM.docx]

**Additional file 1**. **Infant Feeding Styles Questionnaire sub-scale items**

**Restriction with regard to food amount**

It’s important for the parent to decide how much an infant should eat.

It’s important for a parent to have rules about how much a toddler eats.

I carefully control how much (name of child) eats.

I am very careful not to feed (name of child) too much.

**Restriction with regard to food quality**

An infant should never eat fast food.

A toddler should never eat sugary food like candy, ice cream, cake, and cookies.

A toddler should never eat fast food.

A toddler should never eat junk food like potato chips, Doritos, and cheese puffs.

A toddler should only eat healthy food.

I let (name of child) eat junk food like potato chips, Doritos, and cheese puffs. (reverse coded)

I let (name of child) eat fast food. (reverse coded)
