# Supplementary material for: Maternal restrictive feeding and eating in the absence of hunger among toddlers: a cohort study
Source: Int J Behav Nutr Phys Act. 2017 Dec 19;14:172. doi: 10.1186/s12966-017-0630-8 (PMC5735902; doi:10.1186/s12966-017-0630-8)
Supplement: Supplementary file 2 — Unadjusted Pearson correlation coefficients for maternal restrictive feeding and children’s eating in the absence of hunger at 21, 27, and 33 months. (DOCX 55 kb) [file 12966_2017_630_MOESM2_ESM.docx]

| **Additional File 2: Unadjusted Pearson correlation coefficients for maternal restrictive feeding and children’s eating in the absence of hunger at 21, 27, and 33 months** | | | | | | | | | |  |
| --- | --- | --- | --- | --- | --- | --- | --- | --- | --- | --- |
|  | 1 | 2 | 3 | 4 | 5 | 6 | 7 | 8 | 9 | |
| 1. Restriction with regard to amount, 21m | 1.00 | **0.61** | **0.57** | 0.10 | 0.01 | 0.17 | 0.15 | **-0.23** | -0.07 | |
| 2. Restriction with regard to amount, 27m |  | 1.00 | **0.62** | **0.22** | 0.09 | 0.06 | -0.004 | **-0.18** | -0.04 | |
| 3. Restriction with regard to amount, 33m |  |  | 1.00 | 0.13 | -0.004 | 0.15 | **0.26** | -0.03 | 0.02 | |
| 4. Restriction with regard to food quality, 21m |  |  |  | 1.00 | **0.68** | **0.63** | -0.04 | -0.02 | 0.07 | |
| 5. Restriction with regard to food quality, 27m |  |  |  |  | 1.00 | **0.59** | -0.02 | -0.002 | 0.08 | |
| 6. Restriction with regard to food quality, 33m |  |  |  |  |  | 1.00 | 0.12 | 0.13 | 0.05 | |
| 7. EAH, 21m |  |  |  |  |  |  | 1.00 | 0.15 | **0.30** | |
| 8. EAH, 27m |  |  |  |  |  |  |  | 1.00 | **0.41** | |
| 9. EAH, 33m |  |  |  |  |  |  |  |  | 1.00 | |

Note: Correlation coefficients statistically significant at p<.05 are identified in bold
